# A Comparison of a Customized Peripheral Artery Disease (PAD)-Specific Generative AI Chatbot and General-Purpose AI Chatbots for PAD Patient Education

**DOI:** 10.3390/jcm15093317

**Published:** 2026-04-27

**Authors:** Aboubacar Cherif, Megan E. Alagna, Margaret A. Reilly, Lara Lopes, Madison Crutcher, Jennifer Schroeder, Kathryn A. Carey, Anand Brahmandam, David Liebovitz, Karen J. Ho

**Affiliations:** 1 Department of Surgery, Feinberg School of Medicine, Northwestern University, Chicago, IL 60611, USAmegan.alagna@nm.org (M.E.A.); margaret.reilly@nm.org (M.A.R.);; 2 David Geffen School of Medicine at UCLA, Los Angeles, CA 90095, USA; 3 Department of Surgery, Federal University of Minas Gerais, Belo Horizonte 31270-901, MG, Brazil; 4 Department of Medicine, Feinberg School of Medicine, Northwestern University, Chicago, IL 60611, USA

**Keywords:** digital health, artificial intelligence, peripheral artery disease, patient education

## Abstract

**Background/Objective:** Patients with peripheral artery disease (PAD) are known to have poor awareness and understanding of the diagnosis. The role of generative AI chatbots in improving PAD patient education is unknown. Our goal is to compare a generative AI chatbot customized for PAD patient education to publicly available AI chatbots. **Methods:** This is a cross-sectional comparative evaluation of the responses of four AI chatbots to ten prompts that are commonly asked questions about PAD. The three publicly available AI chatbots were ChatGPT-5, Gemini 2.5 Flash, and Claude Sonnet 4.5. We created a customized, voice AI chatbot for PAD education grounded on curated and prompt-injected guidance called Vascular Education and Resources using Artificial Intelligence, or “VERA.” De-identified chatbot-generated responses to inputs were assessed for readability (Flesch–Kincaid Grade Level, Flesch Reading Ease, Gunning Fog Index, Simple Measure of Gobbledygook Index, and Average Reading Level Consensus Score), accuracy, comprehensiveness, and patient education quality (Patient Education Materials Assessment Tool; PEMAT) using validated instruments and expert scoring rubrics. Nonparametric statistical testing was used to compare chatbot performance across all evaluation domains. **Results:** VERA generated the most accessible text compared to the other chatbots and produced responses at a median grade level of 6.6, which was lower than responses from the other chatbots. PAD expert-rated accuracy scores were high across all the chatbots without significant differences between them. Comprehensiveness scores were more varied and demonstrated that VERA was less comprehensive than the other chatbots. PEMAT understandability scores were uniformly high. PEMAT actionability scores were low overall but did not differ significantly across chatbots on post hoc analysis. **Conclusions:** A generative AI chatbot research tool customized for PAD patient education generates textual information about PAD that is more accessible (mean grade level 6.6) than publicly available AI chatbots without loss of accuracy, albeit with modestly reduced comprehensiveness that reflects intentional simplification for patient-centered communication. Future research will assess the acceptability and feasibility of this research tool to be adopted as part of PAD patient education.

## 1. Introduction

Lower extremity peripheral artery disease (PAD) is a highly prevalent [1] manifestation of atherosclerosis that affects blood flow to the legs. The condition impairs walking ability [2] and is associated with increased risk of stroke [3], myocardial infarction [4], and both all-cause and cardiovascular death [4,5]. However, awareness and understanding of PAD is low in both the general public [6,7] and among patients themselves [8,9]. We previously found that patient activation, or the knowledge, skills, and confidence to manage one’s health, is independently associated with PAD knowledge, as assessed by a written survey [10]. Hence, improvements in PAD knowledge among patients may improve health outcomes by increasing engagement in the care plan and improving informed decision-making [11,12]. In a qualitative study, patients with PAD valued interactive discussions for PAD education [13]. In a separate study, PAD providers recognized the importance of patient education and knowledge gaps among patients but described time pressures that preclude adequate direct patient education [14].

Artificial intelligence (AI) has the potential to impact patient education in multiple ways by enabling content delivery that personalizes patient needs, preferences, and learning styles [15]. AI chatbots are computer programs that simulate human conversation and use natural language processing algorithms to understand and respond to user queries [16]. Generative AI-powered chatbots such as ChatGPT, Claude Sonnet, and Google Gemini can also generate new content, such as images and text based on the large language models used to train them [16]. A recent comparison of ChatGPT 4.0 and Gemini 1.0 found varying levels of deficiency in accuracy and readability for both chatbots [17]. A recent study used a custom AI chatbot for diabetes mellitus health literacy and found that responses to prompts were fully appropriate and sourced from the reference knowledge base [18], addressing potential concerns about medical credibility from publicly available chatbots. A separate study of custom AI chatbots for patient education in knee injury found that custom modifications improved accuracy of the information compared to publicly available versions [19]. However, the role of AI in PAD patient education is unknown. Hence, based on our prior qualitative and quantitative research on PAD patient education needs [9,10,13,14], we created a generative AI chatbot customized for PAD patient education as a research tool. The goals of this study are to (1) investigate the ability of publicly available AI chatbots to answer questions about PAD and (2) to compare the customized AI chatbot to publicly available AI chatbots using validated readability and usability metrics and assessments of accuracy and comprehensiveness by PAD experts.

## 2. Materials and Methods

### 2.1. Study Design

This is a cross-sectional comparative evaluation of four AI chatbots as sources of information about PAD. Ten questions (“prompts”) about PAD symptoms, diagnosis, risk factors, and treatment were developed by members of the study team, all of whom are clinicians who routinely treat patients with PAD. The exact wording of each prompt is shown in Table 1. Prompts cover topics that are frequently asked by patients with PAD in our clinical practice or are areas in which patients [9] and the general public [6] have demonstrated knowledge gaps about PAD. Three of the AI chatbots were ChatGPT-5, Gemini 2.5 Flash, and Claude Sonnet 4.5, which are general-purpose, publicly available large language model (LLM) chatbots. The default, publicly available web version of each of these chatbots was used on 28 October 2025. This study did not involve human subjects, patient-level data, or protected health information. As such, institutional review board approval was not required.

### 2.2. Customized Chatbot for PAD Education

We created a generative AI chatbot called Vascular Education and Resources using Artificial Intelligence, or “VERA.” VERA is a research-only education tool designed by the study team to provide evidence-based educational information about PAD in plain language to patients. VERA’s backend was implemented in FastAPI, supporting concurrent multi-user sessions with research identification number authentication, JWT token-based session management, and WebSocket streaming for real-time response delivery. The architecture accommodated multiple LLM providers (OpenAI GPT-4o, GPT-o3-mini, Groq Llama, Groq Gemma); GPT-4o was used exclusively for all study interactions. Speech input was processed through a custom ElevenLabs conversational agent, configured with a research team-selected voice optimized for participant engagement; the pipeline transcribed audio continuously, and audio recordings were discarded immediately upon transcription. Conversation data were kept in a PostgreSQL database. LLM responses were grounded in a curated PAD knowledge base developed from current multi-society guidelines and key literature, curated and reviewed by vascular specialists. System prompts enforced a 5th-grade reading level, empathetic tone, and a structured interaction pattern in which the agent answered the participant’s question, provided a relevant educational fact, and prompted for follow-up questions. Each conversation opened with a standardized plain-language explanation of PAD. The agent dynamically referenced 15 condition-specific educational images (spanning anatomy, diagnostics, treatment, symptoms, and disease progression) displayed in a sidebar for participant review. Safety protocols directed participants describing acute vascular emergencies to seek immediate medical attention, and scope guardrails redirected non-PAD inquiries. Participants can interact via voice or text through an embedded conversational AI widget, with voice calls limited to 20 min. For the purposes of this study, prompts were entered only in text format. The application requires research ID authentication and informed consent acknowledgment, sessions auto-expire after 20 min of inactivity, and all interactions are automatically logged with metadata for transcript analysis. The complete application is publicly available (DOI: 10.5281/zenodo.19042549).

### 2.3. Endpoints

Each prompt was entered as a self-contained query without follow-up interaction to standardize responses across platforms and minimize conversational bias. All chatbot interactions were conducted using default user-facing settings. Publicly available chatbots were accessed through their free, standard interfaces. Each interaction occurred in a new, isolated session to prevent carryover effects from prior prompts. All responses were generated on the same day (28 October 2025) to avoid variations in AI chatbot version within the study. All chatbot responses were recorded, compiled, and anonymized prior to evaluation. De-identified chatbot-generated responses were then assessed for readability, quality (accuracy and comprehensiveness), and patient education quality using validated instruments and expert scoring rubrics. Readability was assessed using the Flesch–Kincaid Grade Level, Flesch Reading Ease, Gunning Fog Index, Simple Measure of Gobbledygook (SMOG) Index, and the Average Reading Level Consensus Score, all of which are validated readability indices [20,21,22]. For ease of calculations, a single online readability calculator was used for readability indices [23]. Results are presented as medians with interquartile ranges for nonparametric analyses, with means and standard deviations reported for descriptive purposes. Accuracy and comprehensiveness were assessed by the PAD experts on the study team (n = 7) using an ordinal scoring system. These experts are American Board of Surgery-certified attending vascular surgeons (n = 2), vascular surgery integrated residents and fellows (n = 3), and advanced practice vascular nurses (n = 2). The experts have been in practice for an average of 12.2 years. Each expert rater was asked to score each response from the perspective of an expert in the diagnosis and management of PAD. In order to reduce bias and inter-rater variability, each expert received the same written instructions on the scoring rubric and worked independently. Accuracy was rated on a 4-point scale, where “1” indicated significant factual problems, “2” indicated partially correct information, “3” indicated mostly correct information, and “4” indicated completely correct information. Comprehensiveness was rated on a 4-point scale, where “1” indicated incomplete information, “2” indicated partially complete information, “3” indicated mostly complete information, and “4” indicated comprehensive information. Patient education quality was assessed using the validated Patient Education Materials Assessment Tool (PEMAT) [24]. PEMAT understandability (when users of diverse backgrounds and varying levels of health literacy can process and explain key messages) and actionability (when users of different backgrounds and varying levels of health literacy can identify what they can do based on the information presented) were scored as percentage values ranging from 0% to 100%, reflecting the proportion of applicable criteria met for each domain [25].

### 2.4. Analysis

Nonparametric statistical testing was used to compare chatbot performance across all evaluation domains. Overall differences in continuous values were assessed using the Kruskal–Wallis test, with Conover–Iman post hoc comparisons performed for pairwise analyses using Holm correction for multiple comparisons. Exploratory prompt-level comparisons were assessed using Friedman tests. Inter-rater agreement was assessed using Fleiss’ kappa. Statistical significance was defined as *p* < 0.05.

## 3. Results

A total of 40 chatbot responses were analyzed, as each of the four chatbots responded to 10 prompts. Outcomes are summarized for readability, expert-rated accuracy and comprehensiveness, and patient education quality metrics. As a representative example of the differences between chatbot responses, the complete chatbot responses for prompt 1 are provided in Supplemental Table S1.

### 3.1. Readability

**Figure 1:**
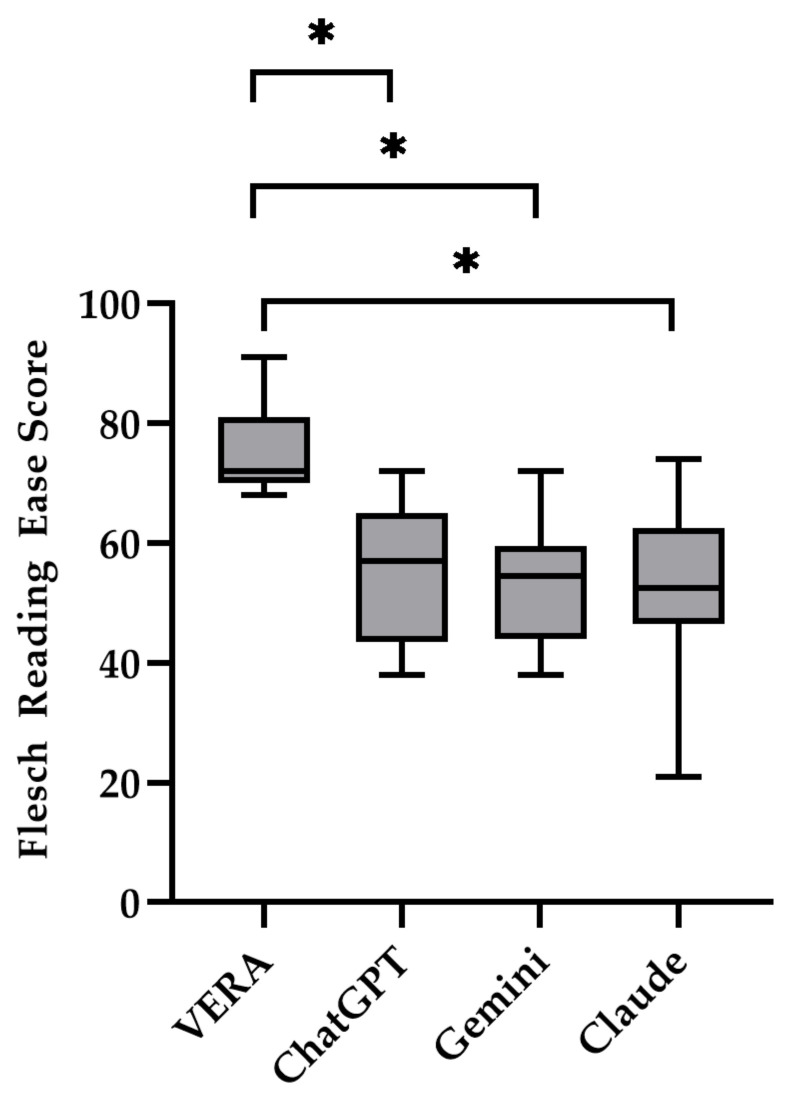
Readability scores by chatbot. Box and whisker plots of Flesch Reading Ease scores for each chatbot. The box borders represent the 25th and 75th percentiles, lines represent the median values, and whiskers represent minimum and maximum values. Scores range from 1–100, with higher scores indicating greater readability. * indicates *p* value < 0.05 (exact values are in Table 2).

Readability differed significantly across the chatbots. VERA consistently generated the most accessible text (Table 2). In terms of the Flesch–Kincaid Grade Level, overall differences were statistically significant (*p* = 0.04). VERA produced responses at a median grade level of 6.6 [4.7, 7.0] compared with 7.5 [6.2, 9.7] for ChatGPT, 9.0 [7.4, 10.2] for Gemini, and 8.0 [6.7, 9.0] for Claude. Pairwise comparisons showed that VERA generated significantly lower grade-level text than Gemini (*p* = 0.01). There was also a statistically significant overall difference in Flesch Reading Ease between chatbots (*p* = 0.0009). VERA achieved the highest readability with a median score of 72 [70, 80], compared to a score of 57 [45, 63] for ChatGPT, 54.5 [44, 58] for Gemini, and 52.5 [47, 62] for Claude. Based on Flesch Reading Ease scores, VERA was significantly more readable than ChatGPT (*p* = 0.001), Gemini (*p* = 0.0003), and Claude (*p* = 0.0006) (Figure 1). Similarly, Gunning Fog Indices differed across the chatbots (*p* = 0.009). VERA produced less complex text with a median score of 75 [6.1, 8.6] compared to Gemini with a score of 10.6 [9.6, 12.3] (*p* = 0.002), and Claude with a score of 10.2 [8.4, 12.0] (*p* = 0.02). VERA generated the lowest median SMOG score of 6.3 [4.6, 6.9] and was significantly less complex than Gemini with a score of 8.8 [7.2, 9.7] (*p* = 0.001). Finally, VERA produced a significantly lower median consensus reading level 7.3 [5.6, 8.3] compared to a score of 10.4 [8.8, 11.4] (*p* = 0.002) for Gemini. According to all readability indices, Gemini and VERA consistently generated the most complex and readable responses, respectively.

### 3.2. Accuracy and Comprehensiveness

Expert-rated accuracy scores were high across models, and exploratory prompt-level comparisons showed no statistically significant differences in accuracy scores between chatbots for any individual prompt (Table 3). Median accuracy scores were predominantly 4 (“completely accurate”) across chatbots for most prompts (Table 4). When accuracy scores were pooled across all prompts, there was a statistically significant overall difference among chatbots (*p* = 0.02; Kruskal-Wallis); however, no pairwise comparisons remained statistically significant after Holm correction (Table 4). Inter-rater variability in expert-rated accuracy scores is further illustrated in scatter plots in Supplemental Figure S1. Inter-rater agreement for accuracy scores was low, with a combined kappa of −0.006.

Within-prompt comparisons of expert-rated comprehensiveness scores demonstrated statistically significant differences among chatbots for 8 of the 10 prompts (Table 5). When comprehensiveness scores were pooled across all prompts, there were marked differences among chatbots (*p* < 0.001; Kruskal–Wallis). Fleiss’ kappa for comprehensiveness ratings was 0.208, indicating modest agreement among raters. Post hoc analyses demonstrated that VERA was significantly less comprehensive than ChatGPT, Gemini, and Claude (all Holm-adjusted *p* < 0.001) (Table 6).

### 3.3. Understandability and Actionability

PEMAT understandability was uniformly excellent across all chatbots. All four models achieved perfect understandability performance (scores of 100%). In contrast, PEMAT actionability scores were low overall and varied across platforms. Mean (SD) actionability scores were 35.0% (23.9) for VERA, 20.0% (28.4) for ChatGPT, 45.0% (19.7) for Gemini, and 40.0% (24.2) for Claude (Table 7). Although PEMAT actionability scores differed significantly across chatbots on overall comparison (Friedman *p* = 0.04), post hoc pairwise Wilcoxon signed-rank tests with Holm correction revealed no statistically significant differences between individual chatbot pairs (Table 8).

## 4. Discussion

In this single-center cross-sectional study, we found that generative AI chatbots can provide accurate information about PAD that is relevant for patients. However, a generative AI chatbot specifically designed for PAD patient education (“VERA”) delivered content at a significantly more accessible reading level compared with publicly available general-purpose models. These findings suggest that domain-specific AI chatbots may offer advantages for patient education by delivering information that is appropriate for patients while maintaining acceptable levels of accuracy.

Public awareness and utilization of generative AI models have increased rapidly over recent years. In February 2026, OpenAI reported that ChatGPT has 900 million weekly users and 50 million paying subscribers [26], while the Google Gemini app had more than 750 million monthly active users [27] and the Claude website had 176.1 million monthly visitors [28]. As patients increasingly turn to these online tools to obtain medical information, understanding the quality and accessibility of chatbot-generated health information is crucial. Indeed, recent prior studies evaluating generative AI chatbots across multiple medical specialties have found that while these models generally provide accurate and organized information, they can be sources of misinformation (AI “hallucinations”) or often generate content that is complex and exceeds the recommended readability standards of sixth to eighth grade for patient education materials [29,30,31,32,33]. In concurrence with our findings, a recent comparison of ChatGPT 4.0 and Gemini 1.0 for PAD education found that both chatbots provided responses to the Cleveland Clinic’s frequently asked questions on PAD that were well above the readability recommendations for patient education materials [17]. This concern is particularly relevant for conditions such as PAD, where the role of AI chatbots for patient education is not yet well defined.

Our prior work has shown that patients with PAD have poor awareness of their diagnosis, which is comparatively less than their awareness of diagnoses of other cardiovascular diseases and PAD risk factors, such as hypertension and diabetes mellitus [9]. PAD-specific knowledge among affected patients is also low [8,9,34,35,36,37,38,39]. In a qualitative study of patients with PAD who watched a PAD video education tool, participants reported a desire to be able to ask questions after the video and to talk to their providers for education [13]. PAD providers who also watched the video noted that even patients with longstanding PAD have significant knowledge gaps [14]. While they preferred to educate their patients directly rather than having them watch or read an educational material, they recognized that time pressures often preclude their efforts at providing direct patient education [14]. Thus, many felt the PAD video education tool was valuable as an educational adjunct rather than a primary source of information. We thus hypothesized that a generative AI chatbot that is customized to provide educational information in an interactive and humanistic manner could address all these patient and provider-identified needs. This approach has also been tested successfully for patient education in diabetes mellitus [18].

VERA is customized, or differs from publicly available chatbots, in several ways. It was trained using sources selected by PAD experts to ensure accuracy and relevance of the information. This is important because publicly available chatbots have been found to provide erroneous medical information. In a study of ChatGPT’s responses to prompts about national cancer guidelines, 34.3% of the responses were partially non-concordant with guidelines and 12.5% of the responses were hallucinated (i.e., not part of any treatment recommendation) [40]. In a different study of the performance of six LLMs in clinical prompts, hallucination rates ranged from 50–82% [41]. It will be important for future iterations of VERA to be subjected to frequent safety evaluations and evidence reviews to ensure that the information provided remains relevant and current. As 21.3% of patients with PAD have marginal or inadequate health literacy [10], VERA was designed to deliver information at approximately a 5th grade literacy level. It also has safeguards in place to ensure that users understand that VERA is not intended to replace a clinician, should not be used for medical emergencies, and that private and identifying information should not be shared.

Prior studies have found that low socioeconomic status, education level, and health literacy are risk factors for health information overload [42] and these are all factors existing in the PAD population [9,10]. Hence, VERA is designed to deliver information in short sentences (which increases readability) and short paragraphs (to avoid information overload). At the end of each response, VERA inquires if additional details or clarification are desired, thus allowing the user to establish the pace or depth of education. However, an important tradeoff of readability of the responses without follow-on prompts is informational depth (comprehensiveness). Although VERA presented information in the most readable or accessible text, expert reviewers found its responses to be less comprehensive than those generated by the general-purpose models. This reflects the inherent challenge of simplifying complex medical concepts while preserving adequate detail about disease mechanisms, risk factors, and treatment options. Whether patients with PAD will find this style of information delivery to be preferable needs further study. However, it is likely that patient education delivery that is tailored to the individual user’s health literacy and learning style will lead to greater knowledge gains than non-tailored approaches [43]. Our findings in this study support further research into the acceptability and feasibility of VERA in PAD patient education. Future work will also include further refinement of VERA to enhance its effectiveness, ensuring that accessibility does not compromise summative comprehensiveness, that the delivery is patient-centered, and that retention and application of the information are optimized.

Another notable observation of the current study is the discrepancy between high understandability and low actionability scores on the PEMAT. All chatbots produced responses that were easily understandable according to PEMAT criteria; however, the responses often lacked specific behavioral guidance or actionable recommendations. This pattern has been observed in prior evaluations of other patient education materials and suggests that educational content frequently focuses on explaining disease processes rather than providing clear instructions for patient action [44] or explaining the applications for daily life. In a survey of internet sites providing information on limb preservation, 99% of patient education materials studied had actionability scores less than 70% [45]. Low actionability responses from all the chatbots in this study may also reflect the nature of the prompts, which asked for explanatory information rather than behavioral recommendations. Thus, the chatbots do not provide suggestions for turning information into tasks or behaviors without being asked explicitly to do so in follow-on prompts. Future customization of VERA can improve actionability by voluntarily incorporating structured guidance for users, such as lifestyle modifications, symptom monitoring, and “when to call” their providers.

This study has several limitations. First, given the rapid evolution of generative AI technology, it is possible that the responses generated by the publicly available chatbots in October 2025 have been updated by the time of publication and thus may affect the reproducibility of our findings. Second, we used only 10 prompts to compare the chatbots; a higher number might have allowed for better differentiation between them and provided a more accurate representation of the real-world patient experience. In other published observational AI chatbot studies, investigators have used a wide range of prompts to compare AI chatbot quality and readability, from 10 [46] to 100 [19]. Third, assessments of accuracy and comprehensiveness were based on expert ratings, which inherently include inter-observer variability, subjectivity, and clinical judgement. The modest Fleiss’ kappa coefficient highlights this variability. Hence, to improve generalizability, we used a scoring rubric to standardize scoring, increased the number of reviewers to 7, included PAD experts from both surgical and nursing backgrounds, and required that the experts work independently. Since all responses were evaluated using the same raters, comparisons between chatbots were internally consistent. Bias was also reduced by deidentifying the chatbot responses prior to grading. Fourth, this study did not assess the impact of follow-on prompts on comprehensiveness, understandability, or usability, or the effect of other VERA features, including voice and graphics, on the user experience, since they detract from a direct comparison with the publicly available chatbots. These features will be the focus of future implementation studies.

## 5. Conclusions

In conclusion, the present study demonstrates that ChatGPT, Gemini, Claude, and VERA can all generate appropriate responses to prompts that are considered frequently asked questions among patients with PAD. However, VERA’s customized delivery of information may be more accessible for the PAD population with inherent tradeoff in comprehensiveness. Future work will focus on further refinements to make VERA more effective as an educational tool and understanding how to best implement it in the clinical context.

## Figures and Tables

**Table 1:** Chatbot prompts.

Prompt	
Q01	What is PAD?
Q02	What are the warning signs of PAD?
Q03	How can I be tested for PAD?
Q04	What medicines are used to treat PAD?
Q05	What surgery options are there for PAD?
Q06	What causes PAD?
Q07	What if my legs hurt when I exercise?
Q08	How serious is PAD?
Q09	What happens if PAD is not treated?
Q10	Do people with PAD have higher risk of stroke and heart attack?

**Table 2:** Readability outcomes by chatbot.

Metric	Chatbot	Median	*p* Value			
			Overall	vs. ChatGPT	vs. Gemini	vs. Claude
Flesch–Kincaid Grade Level	VERA	6.6 (4.7, 7.0)	0.04 *	0.2	0.01 *	0.2
	ChatGPT	7.5 (6.2, 9.7)			0.6	1
	Gemini	9.0 (7.4, 10.2)		0.6		0.8
	Claude	8 (6.7, 9.0)		1	0.8	
Flesch Reading Ease	VERA	72 (70, 80)	0.0009 *	0.001 *	0.0003 *	0.0006 *
	ChatGPT	57 (45, 63)			1	1
	Gemini	54.5 (44, 58)		1		1
	Claude	52.5 (47, 62)		1	1	
Gunning Fog Index	VERA	7.5 (6.1, 8.6)	0.009 *	0.07	0.002 *	0.02 *
	ChatGPT	9.3 (7.4, 11.8)			0.6	1
	Gemini	10.6 (9.6, 12.3)		0.6		1
	Claude	10.2 (8.4, 12.0)		1	1	
SMOG Index	VERA	6.3 (4.6, 6.9)	0.007 *	0.5	0.001 *	0.3
	ChatGPT	7.2 (5.8, 7.7)			0.0498 *	1
	Gemini	8.8 (7.2, 9.7)		0.0498 *		0.08
	Claude	7.3 (6, 7.74)		1	0.08	
Average Reading Level Consensus Score	VERA	7.3 (5.6, 8.3)	0.01 *	0.07	0.002 *	0.05
	ChatGPT	8.6 (7.2, 10.8)			0.6	1
	Gemini	10.4 (8.8, 11.4)		0.6		0.6
	Claude	9.5 (8.2, 10.5)		1	0.6	

SMOG, Simple Measure of Gobbledygook. * indicates *p* < 0.05.

**Table 3:** Prompt-level expert-rated accuracy.

	VERA		ChatGPT		Gemini		Claude		Friedman *p* Value
Prompt	Median [Q1, Q3]	Mean ± Std. Dev.	Median [Q1, Q3]	Mean ± Std. Dev.	Median [Q1, Q3]	Mean ± Std. Dev.	Median [Q1, Q3]	Mean ± Std. Dev.	
Q01	4 [4, 4]	3.86 ± 0.38	4 [4, 4]	3.86 ± 0.38	4 [3.5, 4]	3.57 ± 0.79	4 [3.5, 4]	3.71 ± 0.49	0.8
Q02	4 [3.5, 4]	3.57 ± 0.79	4 [4, 4]	3.86 ± 0.38	4 [4, 4]	3.86 ± 0.38	4 [4, 4]	3.86 ± 0.38	0.8
Q03	4 [3.5, 4]	3.43 ± 1.13	4 [4, 4]	3.86 ± 0.38	4 [4, 4]	3.86 ± 0.38	4 [4, 4]	3.71 ± 0.76	0.8
Q04	2 [2, 4]	2.86 ± 1.07	4 [4, 4]	3.86 ± 0.38	4 [3.5, 4]	3.57 ± 0.79	3 [3, 3.5]	3.14 ± 0.69	0.1
Q05	4 [2, 4]	3.14 ± 1.07	4 [3.5, 4]	3.71 ± 0.49	4 [3.5, 4]	3.71 ± 0.49	3 [3, 3.5]	3.29 ± 0.49	0.4
Q06	4 [2.5, 4]	3.29 ± 0.95	4 [3, 4]	3.57 ± 0.53	4 [3, 4]	3.57 ± 0.53	4 [3, 4]	3.57 ± 0.53	1.0
Q07	4 [2.5, 4]	3.29 ± 0.95	4 [3.5, 4]	3.57 ± 0.79	4 [3, 4]	3.43 ± 0.79	3 [3, 4]	3.29 ± 0.76	0.9
Q08	3 [2, 4]	2.86 ± 1.21	4 [3.5, 4]	3.71 ± 0.49	4 [3.5, 4]	3.71 ± 0.49	4 [3, 4]	3.43 ± 0.79	0.4
Q09	4 [3, 4]	3.43 ± 0.79	4 [4, 4]	3.86 ± 0.38	4 [4, 4]	3.86 ± 0.38	3 [3, 4]	3.43 ± 0.53	0.2
Q10	4 [3, 4]	3.29 ± 1.11	4 [3, 4]	3.43 ± 0.79	4 [3, 4]	3.43 ± 0.79	4 [3.5, 4]	3.71 ± 0.49	0.9

Q, quartile. Std. dev., standard deviation.

**Table 4:** Accuracy score analysis with pooled prompts.

Chatbot	Pooled Across Prompts	Holm-Adjusted *p* Value		
		ChatGPT	Gemini	Claude
VERA	0.02 *	0.06	0.2	1.0
ChatGPT			0.5	0.1
Gemini				0.3

* indicates *p* < 0.05.

**Table 5:** Prompt-level expert-rated comprehensiveness.

	VERA		ChatGPT		Gemini		Claude		Friedman *p* Value
Prompt	Median [Q1, Q3]	Mean ± Std. Dev.	Median [Q1, Q3]	Mean ± Std. Dev.	Median [Q1, Q3]	Mean ± Std. Dev.	Median [Q1, Q3]	Mean ± Std. Dev.	
Q01	2 [1.5, 2]	1.86 ± 0.69	4 [4, 4]	4.00 ± 0.00	3 [2, 3]	2.71 ± 0.76	3 [3, 3.5]	3.14 ± 0.69	0.0005 *
Q02	2 [1.5, 2]	1.86 ± 0.69	4 [4, 4]	4.00 ± 0.00	4 [3.5, 4]	3.71 ± 0.49	4 [3.5, 4]	3.71 ± 0.49	0.0002 *
Q03	2 [1, 2]	1.57 ± 0.53	4 [4, 4]	3.86 ± 0.38	4 [4, 4]	3.86 ± 0.38	3 [2.5, 3]	2.71 ± 0.49	0.00004 *
Q04	2 [2, 2]	2.00 ± 0.58	4 [4, 4]	4.00 ± 0.00	4 [3.5, 4]	3.71 ± 0.49	3 [2.5, 4]	3.14 ± 0.90	0.0005 *
Q05	2 [1, 2]	1.57 ± 0.53	4 [4, 4]	3.86 ± 0.38	4 [4, 4]	4.00 ± 0.00	3 [2.5, 3.5]	3.00 ± 0.82	0.0001 *
Q06	2 [2, 2.5]	2.14 ± 0.69	4 [3, 4]	3.43 ± 0.79	4 [3.5, 4]	3.57 ± 0.79	4 [3.5, 4]	3.71 ± 0.49	0.006 *
Q07	2 [1.5, 2]	1.86 ± 0.69	3 [2.5, 3.5]	2.86 ± 1.07	3 [2.5, 4]	3.00 ± 1.15	3 [2, 3]	2.57 ± 0.98	0.15
Q08	2 [1.5, 2.5]	2.14 ± 1.07	4 [3, 4]	3.43 ± 0.79	4 [3.5, 4]	3.71 ± 0.49	3 [2.5, 4]	3.00 ± 1.15	0.04 *
Q09	2 [1.5, 3]	2.29 ± 1.11	4 [4, 4]	3.86 ± 0.38	4 [4, 4]	4.00 ± 0.00	4 [3, 4]	3.57 ± 0.53	0.002 *
Q10	2 [2, 3.5]	2.57 ± 1.13	3 [3, 4]	3.29 ± 0.76	3 [3, 3.5]	3.14 ± 0.69	4 [3.5, 4]	3.71 ± 0.49	0.1

* indicates *p* < 0.05.

**Table 6:** Comprehensiveness score analysis with pooled prompts.

Chatbot	Pooled Across Prompts	Holm-Adjusted *p* Value		
		ChatGPT	Gemini	Claude
VERA	<0.001 *	<0.001 *	<0.001 *	<0.001 *
ChatGPT			0.27	<0.001 *
Gemini				0.02 *

* indicates *p* < 0.05.

**Table 7:** PEMAT understandability and actionability scores.

	Understandability Scores		Actionability Scores		Friedman *p* Value
Chatbot	Mean % (Std. Dev.)	Median %	Mean % (Std. Dev.)	Median %	
VERA	100 (0)	100	35 (26.9)	50	0.04 *
ChatGPT	100 (0)	100	20 (28.4)	0	
Gemini	100 (0)	100	45.0 (19.7)	50	
Claude	100 (0)	100	40 (24.2)	50	

PEMAT, Patient Education Materials Assessment tool. * indicates *p* < 0.05.

**Table 8:** Post Hoc PEMAT actionability score analysis.

Comparison	Uncorrected *p* Value	Holm-Adjusted *p* Value
VERA vs. ChatGPT	0.1	0.6
VERA vs. Gemini	0.3	1.0
VERA vs. Claude	0.6	0.6
ChatGPT vs. Gemini	0.02 *	0.1
ChatGPT vs. Claude	0.04 *	0.2
Gemini vs. Claude	0.6	1.0

* indicates *p* < 0.05.

## Data Availability

Data are available upon reasonable request to corresponding author.

## Supplementary Materials

The following supporting information can be downloaded at: https://www.mdpi.com/article/10.3390/jcm15093317/s1, Figure S1: Inter-rater variability in expert-rated accuracy scores for each chatbot. Seven expert reviewers scored each response for accuracy on a Likert scale (1–4), as described in the Methods. Scatter plots of accuracy scores for each chatbot and prompt are shown. Each point represents a single score. Mean and median scores are shown in Table 3; Table S1: Representative example of chatbot responses. Shown are the responses of each chatbot to prompt 1.
