# Supplementary material for: A Comparison of a Customized Peripheral Artery Disease (PAD)-Specific Generative AI Chatbot and General-Purpose AI Chatbots for PAD Patient Education
Source: J Clin Med. 2026 Apr 27;15(9):3317. doi: 10.3390/jcm15093317 (PMC13163351; doi:10.3390/jcm15093317)
Supplement: Supplementary file 1 [file jcm-15-03317-s001.zip › jcm-4237358-supplementary.pdf]

“A Comparison of a Customized Peripheral Artery Disease (PAD)-Specific Generative AI Chatbot and General-Purpose AI Chatbots for PAD Patient Education”

Aboubacar Cherif, Megan E. Alagna, Margaret A. Reilly, Lara Lopes, Madison Crutcher, Jennifer Schroeder, Kathryn A. Carey, Anand Brahmandam, David Liebovitz, Karen J. Ho

Supplemental Materials:

1. Supplemental Figure S1
2. Supplemental Table S1

Supplemental Figure S1. Inter-rater variability in expert-rated accuracy scores for each chatbot. Seven expert reviewers scored each response for accuracy on a Likert scale (1-4), as described in the Methods. Scatter plots of accuracy scores for each chatbot and prompt are shown below. Each point represents a single score. Mean and median scores are shown in Table 3.

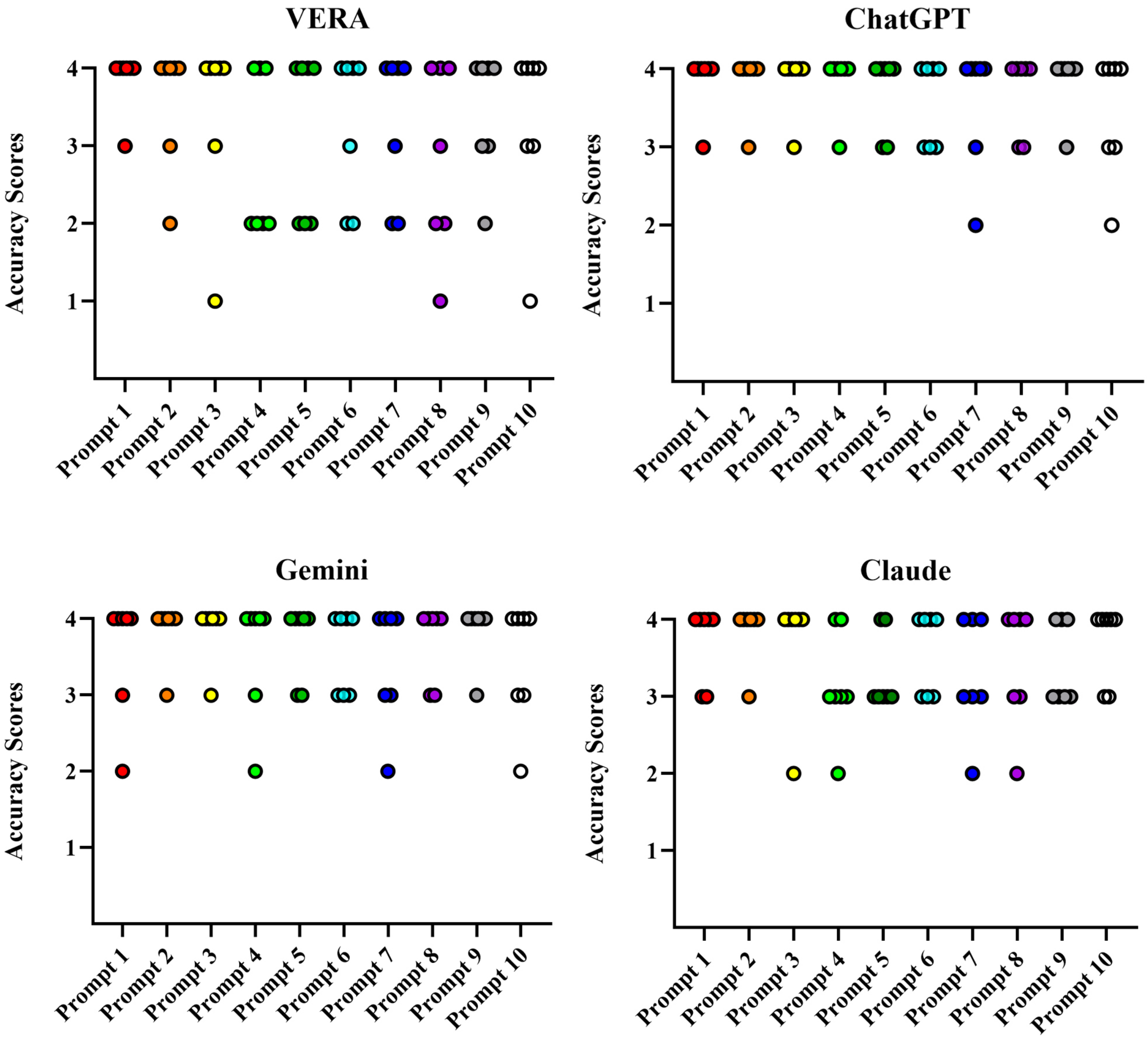

Supplemental Table S1. Representative example of chatbot responses. Shown below are the responses of each chatbot to prompt 1.

Prompt 1	What is PAD?
**VERA**<br>Peripheral artery disease, or PAD, happens when arteries, the blood vessels that carry blood away from your heart, become narrow or blocked. This can make it hard for blood to reach your legs and feet. This might cause leg pain when walking, cold feet, or sores on your legs or feet that don't heal easily. Would you like to know more about what causes PAD or how it can be treated?	
**ChatGPT**<br>Peripheral artery disease (PAD), also called peripheral arterial disease or peripheral vascular disease (PVD), is a common circulatory condition in which narrowed arteries reduce blood flow to the limbs, usually the legs.<br>Here's a breakdown:<br><br>What Happens<br>PAD occurs when fatty deposits (plaque) build up inside the arteries, a process called atherosclerosis. This buildup narrows or blocks arteries, limiting oxygen-rich blood flow to muscles and tissues in the limbs.<br><br>Common Symptoms<br>Many people with PAD have mild or no symptoms, but classic signs include:<br>• Claudication: pain, cramping, or fatigue in the legs or hips during walking or exercise that goes away with rest<br>• Coldness in the lower leg or foot (especially compared to the other side)<br>• Numbness or weakness in the legs<br>• Sores or wounds on the toes, feet, or legs that heal poorly<br>• Change in skin color or shiny skin on the legs<br>• Slow toenail growth or loss of hair on the legs<br>• In severe cases: pain at rest or gangrene<br>•<br>Risk Factors<br>PAD shares many risk factors with heart disease, including:<br>• Smoking (the biggest risk factor)<br>• Diabetes<br>• High blood pressure<br>• High cholesterol<br>• Older age (over 50)<br>• Family history of atherosclerosis<br>•<br>Why It Matters<br>PAD is more than a leg problem; it's a warning sign of widespread atherosclerosis.<br>People with PAD are at much higher risk of heart attack and stroke.<br><br>Diagnosis & Treatment<br>Diagnosis may include:<br>• Physical exam (checking pulses, skin changes)	

- Ankle-brachial index (ABI) test (comparing blood pressure in ankle vs. arm)
- Ultrasound or angiography (imaging blood flow)

Treatment focuses on:

- Lifestyle changes: quitting smoking, regular exercise, healthy diet
- Medications: to improve blood flow, control cholesterol, blood pressure, and diabetes
- Procedures: angioplasty or bypass surgery if blood flow is severely reduced

Gemini

Peripheral artery disease (PAD), also known as peripheral arterial disease, is a common circulatory condition in which narrowed arteries reduce blood flow to the limbs, most often the legs.

The condition is primarily caused by atherosclerosis, which is the buildup of fatty plaque (made of fat, cholesterol, and other substances) on the inside walls of the arteries. This buildup causes the arteries to narrow and stiffen, limiting the flow of oxygen-rich blood to the legs and feet.

Key Features of PAD:

- Location: It typically affects arteries outside of the heart and brain, with the legs being the most common site.
- Cause: Most commonly caused by atherosclerosis (plaque buildup).
- Main Symptom: The classic symptom is claudication, which is painful muscle cramping (most often in the calves, thighs, or buttocks) that begins during physical activity like walking and usually goes away after a few minutes of rest.
- Significance: PAD is often a sign of widespread plaque buildup in the body, meaning people with PAD are at an increased risk for more serious cardiovascular problems, including heart attack and stroke.

If you suspect you or someone you know might have PAD, it is important to consult a healthcare professional. Would you like to know more about the risk factors or treatment options for peripheral artery disease?

Claude

Peripheral artery disease (PAD) is a circulatory condition where narrowed arteries reduce blood flow to your limbs, most commonly the legs. It's usually caused by atherosclerosis, the buildup of fatty deposits (plaque) on artery walls.

Common symptoms include:

- Leg pain or cramping when walking (called claudication) that goes away with rest
- Numbness or weakness in the legs
- Coldness in the lower leg or foot
- Sores or wounds on feet or legs that heal slowly
- Color changes in the legs
- Weak or absent pulse in legs or feet

Risk factors:

- Smoking (the most significant risk factor)
- Diabetes
- High blood pressure
- High cholesterol
- Age over 65
- Family history of PAD or heart disease
- Obesity

PAD is significant because it's not just a leg problem; it often indicates atherosclerosis throughout the body, which increases the risk of heart attack and stroke. Many people with PAD have no symptoms or dismiss their symptoms as normal aging, but it's important to get diagnosed and treated.

Treatment typically involves lifestyle changes (quitting smoking, exercise, healthy diet), medications to manage risk factors and improve blood flow, and in some cases, procedures like angioplasty or bypass surgery to restore circulation.
If you have symptoms or risk factors, it's worth discussing with a doctor, as early detection and treatment can prevent complications and improve quality of life.
